# Nomogram based on clinical characteristics and serological inflammation markers to predict overall survival of oral tongue squamous cell carcinoma patient after surgery

**DOI:** 10.1186/s12903-021-02028-7

**Published:** 2021-12-27

**Authors:** Yi-Wei Lin, Wei-Piao Kang, Bin-Liang Huang, Zi-Han Qiu, Lai-Feng Wei, Biao Zhang, Tian-Yan Ding, Yun Luo, Can-Tong Liu, Ling-Yu Chu, Hai-Peng Guo, Yi-Wei Xu, Yu-Hui Peng

**Affiliations:** 1 Department of Clinical Laboratory Medicine, The Cancer Hospital of Shantou University Medical College, Shantou, 515041 Guangdong People’s Republic of China; 2 Precision Medicine Research Center, Shantou University Medical College, Shantou, 515041 Guangdong People’s Republic of China; 3 Department of Otolaryngology, The Second Affiliated Hospital of Shantou University Medical College, Shantou, 515041 Guangdong People’s Republic of China; 4 Department of Head and Neck Surgery, The Cancer Hospital of Shantou University Medical College, Shantou, 515041 Guangdong People’s Republic of China; 5 Guangdong Esophageal Cancer Institute, The Cancer Hospital of Shantou University Medical College, Shantou, 515041 Guangdong People’s Republic of China; 6 Department of Otolaryngology-Head and Neck Surgery, The First Affiliated Hospital of Shantou University Medical College, Shantou, 515041 Guangdong People’s Republic of China

**Keywords:** Oral tongue squamous cell carcinoma, Inflammation markers, Pretreatment nomogram, Prognosis, Surgery

## Abstract

**Background:**

Oral tongue squamous cell carcinoma (OTSCC) is a prevalent malignant disease that is characterized by high rates of metastasis and postoperative recurrence. The aim of this study was to establish a nomogram to predict the outcome of OTSCC patients after surgery.

**Methods:**

We retrospectively analyzed 169 OTSCC patients who underwent treatments in the Cancer Hospital of Shantou University Medical College from 2008 to 2019. The Cox regression analysis was performed to determine the independent prognostic factors associated with patient’s overall survival (OS). A nomogram based on these prognostic factors was established and internally validated using a bootstrap resampling method.

**Results:**

Multivariate Cox regression analysis revealed the independent prognostic factors for OS were TNM stage, age, lymphocyte-to-monocyte ratio and immunoglobulin G, all of which were identified to create the nomogram. The Akaike Information Criterion and Bayesian Information Criterion of the nomogram were lower than those of TNM stage (292.222 vs. 305.480; 298.444 vs. 307.036, respectively), indicating a better goodness-of-fit of the nomogram for predicting OS. The bootstrap-corrected of concordance index (C-index) of nomogram was 0.784 (95% CI 0.708–0.860), which was higher than that of TNM stage (0.685, 95% CI 0.603–0.767, *P* = 0.017). The results of time-dependent C-index for OS also showed that the nomogram had a better discriminative ability than that of TNM stage. The calibration curves of the nomogram showed good consistency between the probabilities and observed values. The decision curve analysis also revealed the potential clinical usefulness of the nomogram. Based on the cutoff value obtained from the nomogram, the proposed high-risk group had poorer OS than low-risk group (*P* < 0.0001).

**Conclusions:**

The nomogram based on clinical characteristics and serological inflammation markers might be useful for outcome prediction of OTSCC patient.

## Introduction

Oral tongue squamous cell carcinoma (OTSCC) is one of the most prevalent malignancy of the oral cavity worldwide with a relatively poor prognosis [1]. High rates of local recurrence and cervical lymph node metastasis are the most notorious clinical behaviors of OTSCC, which usually cause impairment of patient’s speech, mastication and deglutition [2, 3]. The 5-year survival rate of OTSCC patients is still unsatisfactory even with combined treatments involving surgery, radiotherapy and chemotherapy [4, 5]. Owing to the diverse clinical pathological characteristics of patients, it is important to predict the outcome of OTSCC patients for the selection of more personalized treatment strategies.

At present, the TNM staging system is the gold standard for prognostication in oncology, but it still has limitations. One of the primary disadvantages is its inability to incorporate other variables, such as genetic differences and patient characteristics including age, gender and race, to predict prognosis of cancer patients [6]. Moreover, identifying clinically significant and inexpensive-to-measure prognostic factors obtained before surgery would provide more valuable insights to help clinicians and OTSCC patients to choose appropriate treatment strategy. Hence, it is necessary to identify a robust prognostic tool that can integrate these potential prognostic factors to complement the TNM staging system to better predict the outcome of OTSCC patients. Nomogram is a reliable, user-friendly and sophisticated statistical prediction tool, with the ability to estimate individualized risk via incorporating variables based on the patient and disease characteristics [7]. Nomograms have been widely used for estimating recurrence [8, 9], specific survival [10, 11], overall survival [12, 13] of tumor patients, and may assist clinicians in making individual treatment strategies [6].

Systemic inflammation has been reported to play an important role in the pathogenesis and progression of cancer [14]. Moreover, the association between serological inflammation markers and prognosis of human malignancies has been reported. For example, higher pre-treatment lymphocyte-to-monocyte ratio (LMR) have been shown to be associated with a better prognosis in various tumors [15–18]. And the lower immunoglobulin G (IgG) levels predicted an elevated risk of developing pancreatic cancer compared to the reference levels [19]. Thus, the current study aimed to establish a nomogram to predict OTSCC patient’s outcome based on clinical characteristics and serological inflammation markers which are easy to obtain from routine admission laboratory tests, and assessed the performance of the nomogram with internal validation using a bootstrap resampling method.

## Materials and methods

### Study population

**Fig. 1:**
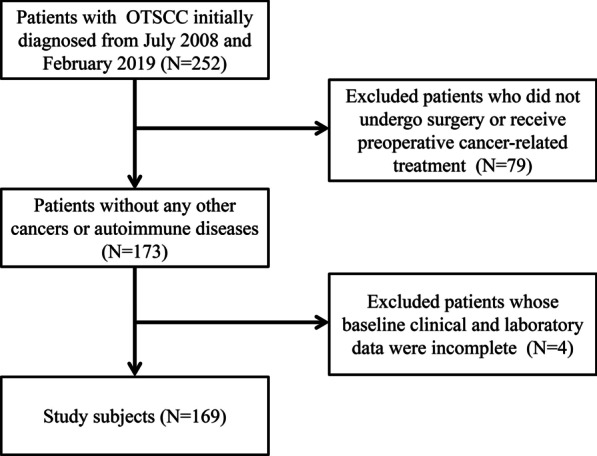
The flowchart for selection procedure of oral tongue squamous cell carcinoma patients

This retrospective study consisted of 169 patients with pathologically-proven OTSCC in the Cancer Hospital of Shantou University Medical College between July 2008 and February 2019 (Fig. 1). Patients included in this study met the following criteria: (1) Tumors were histologically confirmed to be OTSCC. (2) All patients received primary surgical resection but had not undergone preoperative cancer-related treatment. (3) Patients who suffered from any cancers or autoimmune diseases before OTSCC diagnosis were excluded from this study. (4) All patients had complete baseline clinical information and follow-up data. The median follow-up time of patients was 50 months, and the minimum and maximum follow-up time was 1 month and 130 months, respectively. The overall survival (OS) was defined as the interval from the initial diagnosis to either any form of death or the last follow-up time. The last follow-up was performed in September 2019. This study was approved by the Hospital Ethics Committee in Cancer Hospital of Shantou University Medical College and informed consents were obtained from all included participants. All work was complied with the principles of the Helsinki Declaration.

Clinical baseline data of each patient was collected as follows: clinical pathological TNM stage, gender, age and tumor size. Tumor stage were classified according to the eighth edition of the Union for International Cancer Control/American Joint Cancer Committee (AJCC) TNM staging system [20]. Peripheral blood samples of the patients were collected before surgery began. The potential serum prognostic factors included IgG, immunoglobulin A (IgA), immunoglobulin M (IgM), Complement 3 (C3), Complement 4 (C4), B factor (BF), C-reactive protein (CRP), white blood cell count (WBC), platelet-to-lymphocyte ratio (PLR), neutrophil-to-lymphocyte ratio (NLR) and LMR.

### Model construction and assessment

In this study, continuous variables were transformed into categorical variables and the optimal cut-off values for the continuous variables were obtained by X-tile [21]. Prognostic factors for OS were selected by Cox proportional hazards regression analysis, and those with a significant level of *P* ≤ 0.10 in univariate analysis were analyzed using multivariate Cox regression analysis. A nomogram with endpoints of 1-, 3- and 5-year OS was constructed based on the multivariate analysis results. By comparing with selected prognostic factors and TNM stage, the goodness-of-fit and discriminative ability of the nomogram were examined with Akaike Information Criterion (AIC) and Bayesian Information Criterion (BIC), and concordance index (C-index), respectively. The calibration of the nomogram was assessed with calibration curve, and decision curve analysis was conducted to estimate the clinical utility of the nomogram. The Kaplan–Meier method and log-rank test were applied for calculating and comparing the differences in OS. All internal validations were performed using bootstrapping method with 1,000 resamples.

### Statistical analyses

Statistical analyses were performed using SPSS software, version 19.0 (SPSS Inc., Chicago, IL, USA) and R (version 4.0.2) for Windows. The nomogram, decision curve analysis curves and calibration curves were plotted by the *rms* package in R. Time-dependent C-index curves were plotted by the *pec* package in R. Survival curve was plotted using Kaplan–Meier survival analysis and compared using the log-rank test with the *survminer* and *survival* package in R.* P* < 0.05 was considered statistically significant.

## Results

### Patient characteristics

We recruited 169 OOTSCC patients with complete baseline clinical and laboratory data (Fig. 1). The clinical characteristics of these patients were shown in Table 1. The median age for patients was 57 years (range 25–88 years), of which 93 (55%) were males and 76 (45%) were females. The numbers of patient with I–II and III–IV stage were 147 (87%) and 22 (13%), respectively. The optimal cut-off values for the continuous variables were obtained by X-tile as follows: age (69 y), IgG (12.51 g/L, range 6.06–49.02), IgA (1.97 g/L, range 0.48–4.58), IgM (1.09 g/L, range 0.32–3.61), C3 (1.05 g/L, range 0.42–2.05), C4 (0.22 g/L, range 0.097–0.516), BF (0.47 g/L, range 0.2–0.92), CRP (3.78 mg/L, range 0.55–69.39), WBC (4.95 × 10^9^/L, range 3.1–12.2), LMR (4.15, range 1.36–10.2), NLR (1.61, range 0.44–7.7) and PLR (177.86, range 36.98–548.86).

**Table 1:** Demographics and clinical characteristics of OTSCC patients

Characteristics	No. of Patients (N = 169), n (%)	No. of Events (N = 35), n (%)
Gender		
Male	93 (55)	19 (54)
Female	76 (45)	16 (46)
Age (years)		
< 69	141 (84)	27 (77)
≥ 69	28 (16)	8 (23)
Tumor size (cm)		
< 4.2	150 (89)	26 (74)
≥ 4.2	19 (11)	9 (26)
TNM stage		
I–II	147 (87)	14 (40)
III–IV	22 (13)	21 (60)
Treatment		
Surgery	121 (72)	14 (40)
Surgery followed by radiotherapy/chemotherapy	48 (28)	21 (60)
IgG (g/L)		
< 12.51	85 (50)	23 (66)
≥ 12.51	84 (50)	12 (34)
IgA (g/L)		
< 1.97	73 (43)	15 (43)
≥ 1.97	96 (57)	20 (57)
IgM (g/L)		
< 1.09	78 (46)	19 (54)
≥ 1.09	91 (54)	16 (46)
C3 (g/L)		
< 1.05	86 (51)	15 (43)
≥ 1.05	83 (49)	20 (57)
C4 (g/L)		
< 0.22	56 (33)	11 (31)
≥ 0.22	113 (67)	24 (69)
BF (g/L)		
< 0.47	142 (85)	29 (83)
≥ 0.47	27 (15)	6 (17)
CRP (mg/L)		
< 3.78	143 (85)	28 (80)
≥ 3.78	26 (15)	7 (20)
WBC (× 10^9^/L)		
< 4.95	30 (17)	9 (26)
≥ 4.95	139 (83)	26 (74)
PLR		
< 177.86	148 (88)	29 (83)
≥ 177.86	21 (12)	6 (17)
NLR		
< 1.61	63 (37)	16 (46)
≥ 1.61	106 (63)	19 (54)
LMR		
< 4.15	96 (57)	27 (77)
≥ 4.16	73 (43)	8 (23)

TNM, tumor/node/metastasis; OTSCC, oral tongue squamous cell carcinoma; IgG, immunoglobulin G; IgA, immunoglobulin A; IgM, immunoglobulin M; C3, Complement 3; C4, Complement 4; BF, B factor; CRP, C-reactive protein; WBC, white blood cell count; PLR, platelet-to-lymphocyte ratio; NLR, neutrophil-to-lymphocyte ratio; LMR, lymphocyte-to-monocyte ratio

### Construction of the nomogram based on clinical and serological markers

**Fig. 2:**
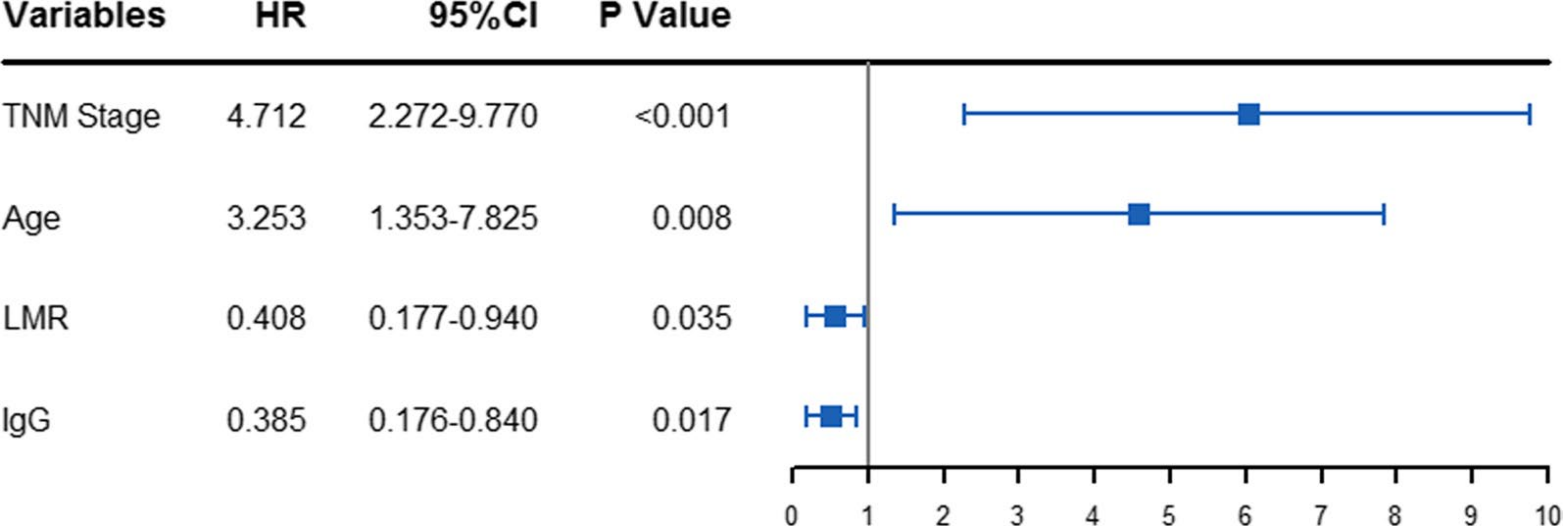
The HR and 95% CI of four independent prognostic factors for OS. TNM, tumor/node/metastasis; LMR, lymphocyte-to-monocyte ratio; IgG, immunoglobulin G; HR, hazard ratio; 95% CI, 95% confidence interval; OS, overall survival

The univariate and multivariate Cox analysis were used to select the potential prognostic markers, and estimate their influence on OS for OTSCC patients. The multivariate analysis results showed that the following variables remained significantly independent prognostic factors: TNM stage (*P* < 0.001, HR = 4.712; 95% CI 2.272–9.770), age (*P* = 0.008, HR = 3.253; 95% CI 1.353–7.825), LMR (*P* = 0.035, HR = 0.408; 95% CI 0.177–0.940) and IgG (*P* = 0.017, HR = 0.385; 95% CI 0.176–0.840) (Fig. 2). The detailed results of univariate and multivariate analyses were presented in Table 2.

**Table 2:** Univariate and multivariate Cox proportional hazards regression analysis for OS

	Univariate analysis			Multivariate analysis		
	HR	95%CI	*P*	HR	95%CI	*P*
Gender (female vs. male)	1.099	0.565–2.137	0.782			
Age (≥ 69 vs. < 69; y)	1.997	0.905–4.408	0.087	3.253	1.353–7.825	0.008
Tumor size (≥ 4.2 vs. < 4.2; cm)	2.945	1.376–6.304	0.005			
TNM Stage (III–IV vs. I–II)	4.396	2.230–8.664	0.000	4.712	2.272–9.770	0.000
IgG (≥ 12.51 vs. < 12.51; g/L)	0.457	0.225–0.926	0.030	0.385	0.176–0.840	0.017
IgA (≥ 1.97 vs. < 1.97; g/L)	1.474	0.773–2.812	0.239			
IgM (≥ 1.09 vs. < 1.09; g/L)	0.538	0.273–1.060	0.073			
C3 (≥ 1.05 vs. < 1.05; g/L)	1.924	0.967–3.830	0.062			
C4 (≥ 0.22 vs. < 0.22; g/L)	1.582	0.773–3.235	0.209			
BF (≥ 0.47 vs. < 0.47; g/L)	2.087	0.876–4.968	0.097			
CRP (≥ 3.78 vs. < 3.78; mg/L)	1.621	0.722–3.638	0.241			
WBC(≥ 4.95 vs. < 4.95; 10^9^/L)	0.467	0.218–1.003	0.051			
LMR (≥ 4.15 vs. < 4.15)	0.243	0.110–0.539	0.001	0.408	0.177–0.940	0.035
PLR (≥ 177.86 vs. < 177.86)	2.205	0.913–5.325	0.079			
NLR (≥ 1.61 vs. < 1.61)	0.682	0.351–1.328	0.261			

HR, Hazard ratio; 95% CI, 95% confidence interval; TNM, tumor/node/metastasis; IgG, immunoglobulin G; IgA, immunoglobulin A; IgM, immunoglobulin M; C3, Complement 3; C4, Complement 4; BF, B factor; CRP, C-reactive protein; WBC, white blood cell count; LMR, lymphocyte-to-monocyte ratio; PLR, platelet-to-lymphocyte ratio; NLR, neutrophil-to-lymphocyte ratio; OS, overall survival

**Fig. 3:**
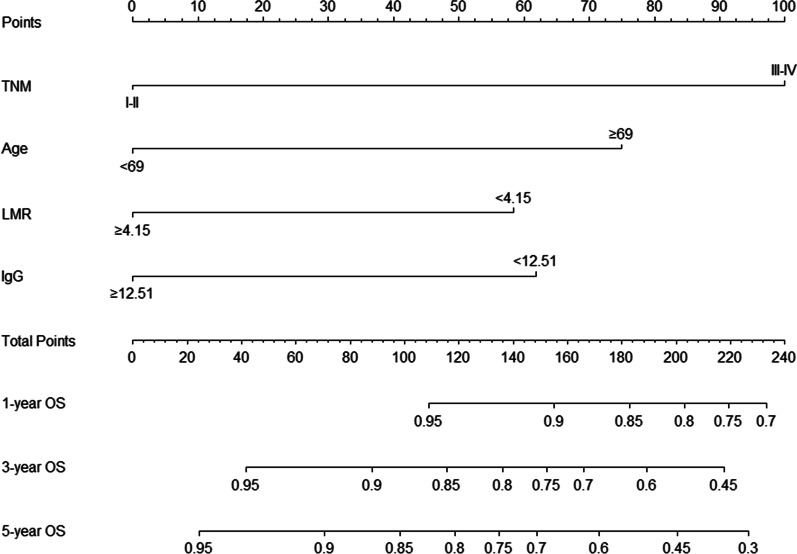
Nomogram based on TNM stage, age, LMR and IgG in prediction for 1-, 3- and 5-year OS of OTSCC patient. The nomogram was used by summing the points identified on the points scale for each prognostic factor. The total points projected on the bottom scales match the probability of 1-, 3-, and 5-year survival of patient. OS, overall survival; LMR, lymphocyte-to-monocyte ratio; IgG, immunoglobulin G; OTSCC, oral tongue squamous cell carcinoma; TNM, tumor/node/metastasis

Incorporating these prognostic markers including TNM stage, age, LMR and IgG, the nomogram was constructed for 1-, 3- and 5-year OS prediction (Fig. 3). From the nomogram, each prognostic factor had a risk point, which could be obtained by drawing a vertical line directly upward from the corresponding value of the prognostic factor to an axis with “Points”. In order to determine the 1-, 3-, and 5-year OS probability of a specific patient, a vertical line could be drawn from the “Total Points” which was the sum of the risk points of all prognostic factors, to the axis marked “1-, 3-, and 5-year OS”. And a larger “Total Points” score would represented a worse OS for the patient.

### The goodness-of-fit and discrimination of the nomogram

**Fig. 4:**
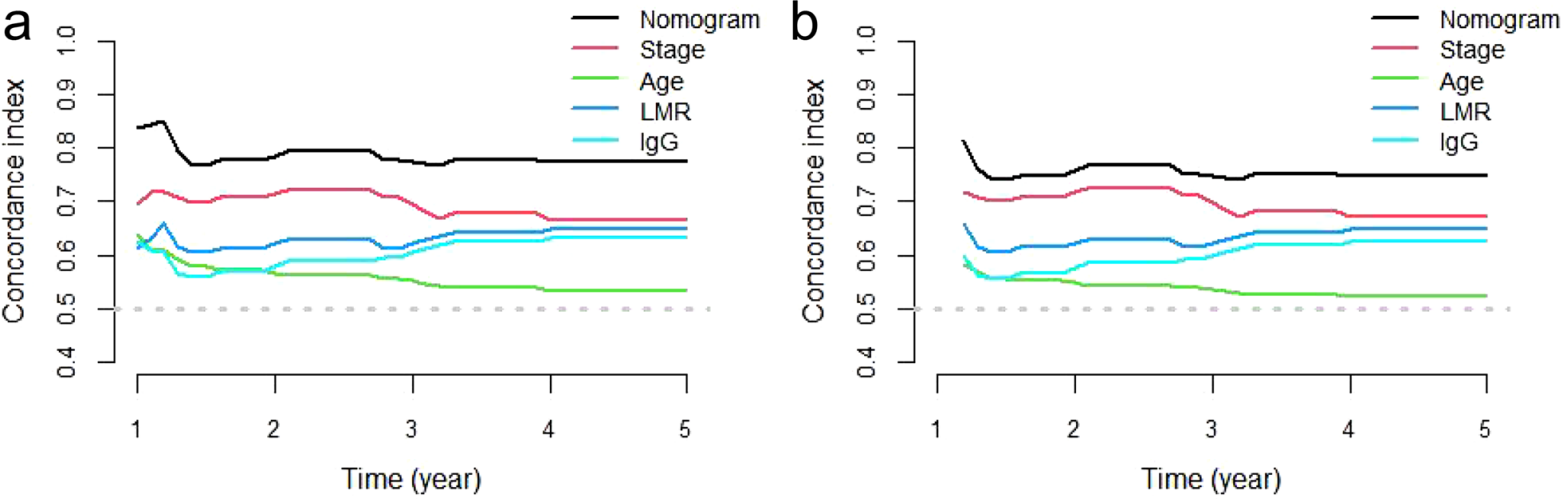
Time-dependent C-index of nomogram compared with TNM stage, age, LMR and IgG for OS of OTSCC patient (**a**) and internally validated with using a bootstrap resampling method (**b**). C-index, concordance index; OS, overall survival; LMR, lymphocyte-to-monocyte ratio; IgG, immunoglobulin G; OTSCC, oral tongue squamous cell carcinoma; TNM, tumor/node/metastasis

The goodness-of-fit and discriminative ability of the nomogram were examined by the AIC and BIC, and C-index, respectively. The results were presented in Table 3. The AIC and BIC of the nomogram for OS were much lower than those of TNM stage (292.222 vs. 305.480; 298.444 vs. 307.036, respectively), indicating that the nomogram had a higher goodness-of-fit for predicting OS. The bootstrap-corrected C-index of the nomogram was 0.784 (95% CI 0.708–0.860), which was higher than that of TNM stage (0.685, 95% CI 0.603–0.767, *P* = 0.017). Moreover, time-dependent C-index analysis also showed that the nomogram model exhibited good prognostic accuracy in clinical outcome prediction either for 1-, 3- and 5-year OS of patient when compared with TNM stage and any single prognostic marker (Fig. 4A). A similar result was also observed in internally validation using a bootstrap resampling method (Fig. 4B).

**Table 3:** The AIC, BIC and C-index of prognostic factors and nomogram for prediction OS

	C-index (95% CI)	*P*-value	AIC	BIC
TNM	0.685 (0.603–0.767)		305.480	307.036
Age	0.556 (0.480–0.632)		321.534	323.089
LMR	0.638 (0.558–0.718)		309.457	311.012
IgG	0.602 (0.518–0.686)		318.899	320.454
Nomogram	0.784 (0.708–0.860)		292.222	298.444
Nomogram versus TNM		0.017		
Nomogram versus Age		< 0.001		
Nomogram versus LMR		< 0.001		
Nomogram versus IgG		< 0.001		

C-index, concordance index; 95% CI, 95% confidence interval; AIC, Akaike Information Criterion; BIC, Bayesian Information Criterion; LMR, lymphocyte-to-monocyte ratio; IgG, immunoglobulin G; TNM, tumor/node/metastasis; OS, overall survival; *P*-values are calculated based on normal approximation using function rcorrp.cens in *Hmisc* package

### Net benefit and predictive capacity of the nomogram

**Fig. 5:**
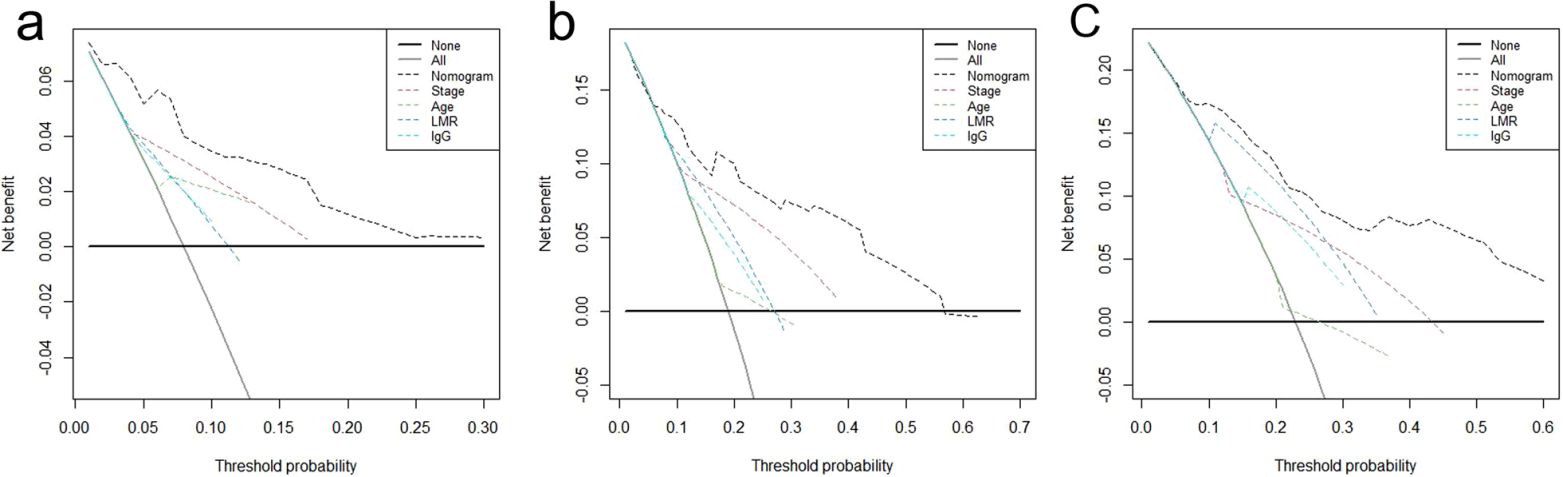
Decision curve analysis of nomogram compared with TNM stage, age, LMR and IgG for 1-year OS (**a**), 3-year OS (**b**), 5-year OS (**c**) of OTSCC patient. The thick grey line is the net benefit for a strategy of treating all men; the thick black line is the net benefit of treating no men. The y-axis indicates the overall net benefit, which is calculated by summing the benefits (true positive results) and subtracting the harms (false positive results). OS, overall survival; LMR, lymphocyte-to-monocyte ratio; IgG, immunoglobulin G; OTSCC, oral tongue squamous cell carcinoma; TNM, tumor/node/metastasis

**Fig. 6:**
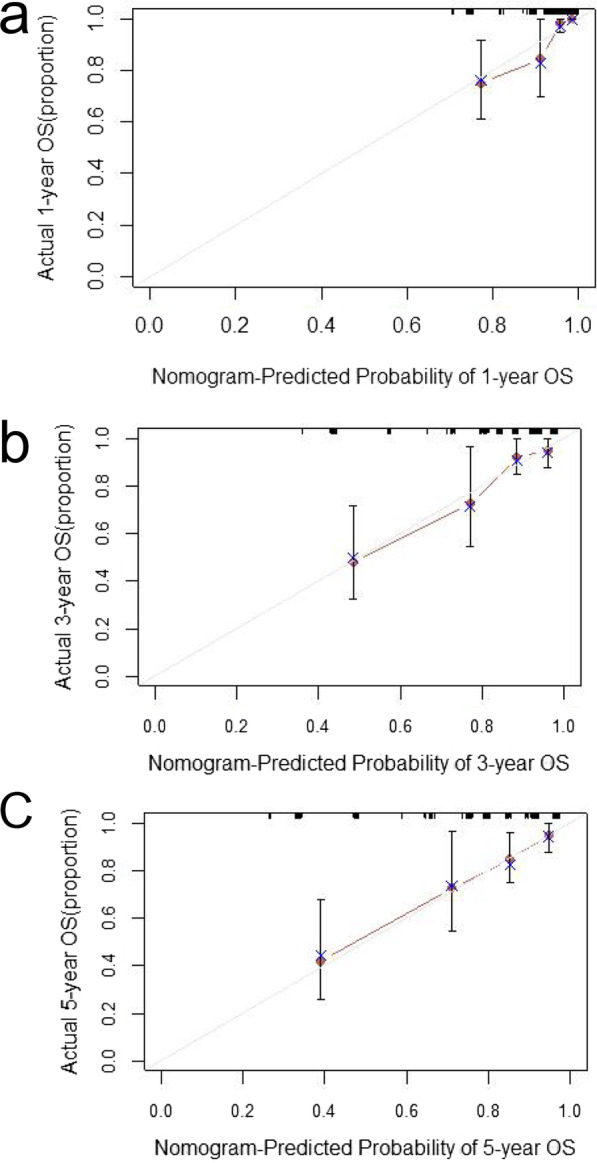
Calibration curves for 1-year OS (**a**), 3-year OS (**b**), 5-year OS (**c**) of nomogram predictions. OS, overall survival

The decision curve analysis and calibration curves were used to determine net benefit and predictive capacity of the nomogram. As shown in Fig. 5, the decision curves analysis for 1-, 3-, and 5-year OS showed that the nomogram had higher overall net benefit compared with traditional TNM stage across the majority of the range of reasonable threshold probabilities. In addition, calibration would estimate how close the nomogram estimated risk was to the observed risk, depicted by a calibration plot. Figure 6 illustrated the good calibration of our nomogram for the 1-, 3-, and 5-year OS predictions. Taken together, these results demonstrated that our nomogram had a better performance to predict survival outcomes of OTSCC patients when compared with TNM stage.

### Risk stratification based on the nomogram

**Fig. 7:**
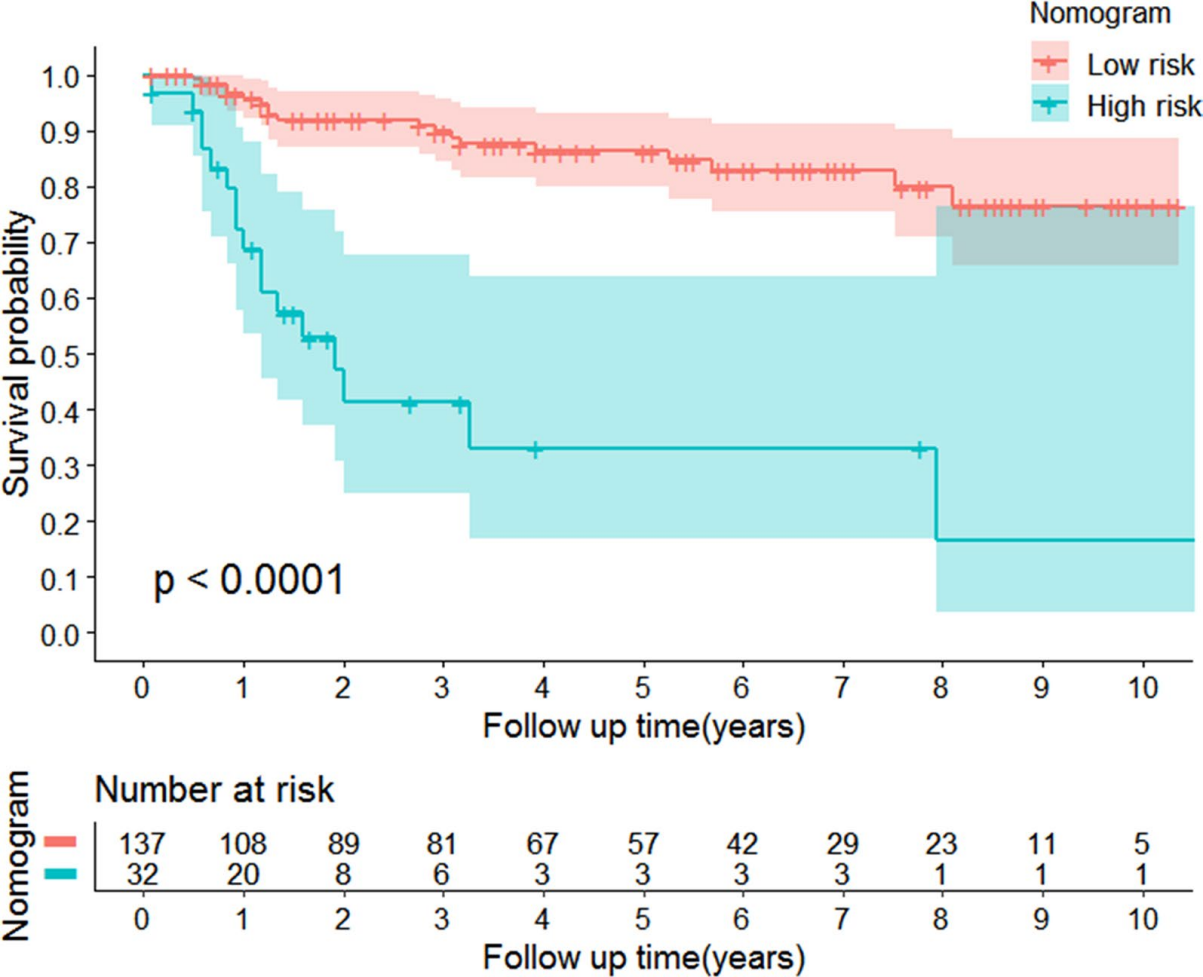
Kaplan-Meier curve for OS based on the prediction of nomogram. Low risk, Total points < 195.13 for OS; High risk, Total points ≥ 195.13 for OS. OS, overall survival

To assess whether the OTSCC patients could be effectively separated into two proposed risk groups based on the nomogram and OS, we calculated each patient’s total point and used the X-tile program to determine the optimal cutoff value. Using the cutoff value of 195.13, the OTSCC patients were subdivided into low- and high-risk groups, and Kaplan-Meier survival analysis was applied to assess their survival. Compared with patients in the low-risk group whose median OS was 47 months, patients in the high-risk group had shorter OS (median OS: 15 months; *P* < 0.0001; Fig. 7).

## Discussion

OTSCC is one of the most aggressive tumors of oral cavity with a relatively poor prognosis [1]. At present, the prognosis and treatment of OTSCC patients are primarily determined by the AJCC TNM staging system. However, even at the same stage of OTSCC, the prognosis of patients is still highly different since it is influenced by a variety of factors [22]. Therefore, in order to find other reliable prognostic factors to complement the TNM stage system to better predict patient’s outcomes and help guide treatments, we used Cox proportional hazards regression analysis to determine TNM stage, age, LMR, IgG as independent prognostic factors for OS. Then, a nomogram that incorporated these four prognostic factors to predict OS of OTSCC patients was established and internally validated. Our nomogram showed enhanced predictive accuracy and discriminative ability when compared with the traditional TNM stage system. Moreover, the nomogram signature successfully separated OTSCC patients into high-risk and low-risk groups with significant differences in OS.

To date, increasing research have indicated a significant link between systemic inflammatory response and progression and prognosis of various types of tumors [23]. It has shown that several pretreatment peripheral indicators of immunity/inflammation are significant factors in predicting the progression and prognosis of tumors [16, 24]. LMR, as one of the immunity/inflammation indicators, was studied for possible correlation with OTSCC patient’s outcome in this study. LMR has been reported to enter into prognostic nomograms for pancreatic [16], colorectal [17], epithelial [18], tongue [25], and other solid cancers [15]. All these studies suggested that a higher LMR was related to favorable prognosis, which was also confirmed in our study (HR = 0.408; 95% CI 0.177–0.940; *P* = 0.035). Although the prognostic value of LMR in tumors seems clear, the actual mechanisms by which it contributes to improve survival outcome require further study. LMR is defined as the absolute peripheral lymphocyte count divided by the absolute peripheral monocyte count. Admittedly, lymphocytes play a critical role in the immune response and destroy residual cancer cells by recognizing tumor antigens [23]. Moreover, Tumor-infiltrating lymphocytes (TILs) are thought to be responsible for cellular as well as humoral anti-tumor immune responses that contribute to suppress tumor proliferation, invasion, and metastasis. Indeed, higher numbers of TILs were associated with better clinical outcomes [26, 27], and lymphopenia was found to be correlated to worse OS in prospectively collected series of patients with metastatic breast cancer, non-Hodgkin lymphoma, and soft tissue sarcoma [28]. On the other hand, a higher monocyte count has been presented as a poor prognostic factor in metastatic melanoma [29] and cervical cancer [30]. Tumor-associated macrophages, which are derived from circulating monocytes and accelerate tumor progression by production of growth factors and cytokines, have also been suggested to be related to unfavorable prognosis in breast cancer [31], and Hodgkin's lymphoma [32]. Thus, a lower LMR may indicate an imbalance of the inflammatory response, which would be reflected by a weak antitumor immunity and favorable microenvironment for tumor growth.

IgG represents a highly abundant antibody subtype in human serum and is a key component in anti-tumor humoral immune response [33]. A number of studies have demonstrated that aberrant post-translational modifications of IgG are responsible for numerous pathological processes including cancer [34–36]. Moreover, the evidence for an inverse association between pre-diagnostic serum IgG level and the risk of developing melanoma or pancreatic cancer was found in the Swedish Apolipoprotein-related MORtality RISk (AMORIS) cohort study. The humoral response might provide a protective role against the development of melanoma or pancreatic cancer, mediated through IgG [19, 37]. In the current study, we revealed that a higher IgG level was not only an independent prognostic factor, but was also associated with better OS in OTSCC patients (HR = 0.385; 95% CI 0.176–0.840; *P* = 0.017). This association may be explained by humoral response, which would play a critical role in suppression of tumor behaviors.

Recently, a wide variety of prognostic nomograms based on patients’ demographics and clinicopathological parameters, such as age, gender, race, tumor site and depth of tumor invasion, have been developed for survival prediction of patients with tongue cancer [2, 38–45]. On the other hand, most studies indicated that single serological inflammation marker, such as CRP [46], NLR [47, 48] and LMR [25], could serve as an independent prognostic factor for OTSCC patients’ survival prediction. Together, these findings might help clinicians to identify patients that would benefit from surgical resection and/or neck dissection strategies or, alternatively, if additional treatment methods need to be explored. In order to comprehensively improve prognostic accuracy and develop a multi-parametric prognostic model, number of potential serological inflammation markers and patients’ demographics and clinicopathological features were included and assessed together in the current study. The results showed that our nomogram based on TNM stage, age, LMR and IgG, had incremental prognostic value compared to the traditional TNM staging system or any single serological inflammation marker. A similar study by Lu et al. also established a nomogram incorporating patients’ clinicopathological factors and serological inflammation marker to predict survival for patients with OTSCC [22]. However, the serological inflammation markers included in Lu et al.’s study were LMR, NLR, PLR and SII(systemic immune-inflammation index), and their nomogram construction was based on age, lymph node density (LND) and SII, which were different from ours. In our study, we collected more serological inflammation markers to evaluate the prognostic value for predicting OTSCC patients’ survival, such as IgG, IgA, IgM, C3, C4 and BF, all of which played an important role in development and progression of cancer, and might have potential prognostic value for cancer patient’s survival prediction [49–56]. Moreover, we identified IgG could served as an independent prognostic factor for survival prediction of OTSCC patients, and higher IgG level was associated with better OS in OTSCC patients.

Taken together, based on the results of Cox regression analysis, our nomogram consisted of four prognostic factors: TNM stage, age, LMR and IgG. The data showed that our nomogram had a better goodness-of-fit for predicting OS of OTSCC patient. And the C-index of the nomogram was much higher than that of TNM stage. Time-dependent C-index analysis also showed that our nomogram exhibited good prognostic accuracy in clinical outcome prediction for OTSCC patient when compared with TNM stage. At the same time, the decision curve analysis and calibration plots of 1-, 3- and 5-year OS showed that the nomogram had higher predictive accuracy and overall net benefit than TNM stage. Moreover, the nomogram could successfully classified OTSCC patients into high-risk and low-risk subgroups, and the high-risk patients had poor survival outcomes. Taken together, it seems that our nomogram could be helpful in predicting the OTSCC patient’s outcome, and in treatment decisions-making for OTSCC patients.

However, there are still some limitations in our study. First, our findings were based on a retrospective design, and thus, this study cannot exclude all potential bias. Second, our data was obtained from a single cancer center, and the sample size was small. A larger sample size from other institutions would be required to further validate our results. Finally, the endpoint of our study was OS, and more research on the disease-free survival should also be carried out in the future.

## Conclusions

This study provided a novel nomogram based on clinical characteristics and serological inflammation markers with satisfactory performance when compared with traditional TNM stage system for individualized OS estimation. In the future, if a large-scale, multicenter prospective validation could be completed, our nomogram may be useful in clinical practice as a simple and readily available prognostic tool.

## Data Availability

The datasets used and/or analysed during the current study are available from the corresponding author on reasonable request.

## Abbreviations

AIC: Akaike Information Criterion
AJCC: American Joint Cancer Committee
BF: B factor
BIC: Bayesian Information Criterion
C3: Complement 3
C4: Complement 4
C-index: Concordance index
CRP: C-reactive protein
HR: Hazard ratio
IgG: Immunoglobulin G
IgA: Immunoglobulin A
IgM: Immunoglobulin M
LMR: Lymphocyte-to-monocyte ratio
NLR: Neutrophil-to-lymphocyte ratio
OS: Overall survival
PLR: Platelet-to-lymphocyte ratio
TILs: Tumor-infiltrating lymphocytes
TNM: Tumor/node/metastasis
OTSCC: Oral tongue squamous cell carcinoma
WBC: White blood cell count
95% CI: 95% Confidence interval
